# Targeting *O*-GlcNAcylation to overcome resistance to anti-cancer therapies

**DOI:** 10.3389/fonc.2022.960312

**Published:** 2022-08-17

**Authors:** Ninon Very, Ikram El Yazidi-Belkoura

**Affiliations:** ^1^ Université de Lille, Inserm, CHU Lille, Institut Pasteur de Lille, U1011-EGID, Lille, France; ^2^ Université de Lille, CNRS, UMR 8576-UGSF-Unité de Glycobiologie Structurale et Fonctionnelle, Lille, France

**Keywords:** 6845/12000 *O*-GlcNAcylation, cancer, therapy resistance, therapeutic strategy, post-translational modifications (PTMs)

## Abstract

In cancer cells, metabolic reprogramming is associated with an alteration of the *O*-GlcNAcylation homeostasis. This post-translational modification (PTM) that attaches *O*-GlcNAc moiety to intracellular proteins is dynamically and finely regulated by the *O*-GlcNAc Transferase (OGT) and the *O*-GlcNAcase (OGA). It is now established that *O*-GlcNAcylation participates in many features of cancer cells including a high rate of cell growth, invasion, and metastasis but little is known about its impact on the response to therapies. The purpose of this review is to highlight the role of *O*-GlcNAc protein modification in cancer resistance to therapies. We summarize the current knowledge about the crosstalk between *O*-GlcNAcylation and molecular mechanisms underlying tumor sensitivity/resistance to targeted therapies, chemotherapies, immunotherapy, and radiotherapy. We also discuss potential benefits and strategies of targeting *O*-GlcNAcylation to overcome cancer resistance.

## Introduction

One of the main hallmarks of cancer cells is energy metabolism reprogramming to support continuous proliferation (1). This mechanism, known as the Warburg effect, shifts energy production from the oxidative phosphorylation that operates in normal cells to the faster aerobic glycolysis. To compensate for the low energy yield of aerobic glycolysis, tumor cells overexpress Glucose Transporters (GLUTs) (2) and increase their glucose uptake by a factor of 10. In addition to glucose, cancer cells excessively consume glutamine as another source of carbon and nitrogen to produce nucleic acids, lipids, and proteins (3). The conversion of glucose into fructose-6-phosphate is a common step in both glycolysis and the hexosamine biosynthetic pathways (HBP), which metabolizes 2 to 3% of total glucose entering the cell (4).

The final product of the HBP is the nucleotide-sugar uridine diphosphate N-acetylglucosamine (UDP-GlcNAc) that requires building blocks produced by glucose, amino acids (mainly glutamine but also glucogenic and ketogenic amino acids), fatty acids (acetyl-coenzyme A, acetyl-CoA) and nucleotides (uridine triphosphate, UTP) metabolisms. UDP-GlcNAc is therefore considered a cellular nutritional sensor. UDP-GlcNAc serves as a substrate of OGT to *O*-GlcNAcylate serine (Ser) and threonine (Thr) residues of cytoplasmic, nuclear, and mitochondrial proteins; the OGA removes it. The activity of both OGT and OGA makes this ubiquitous intracellular post-translational modification highly dynamic as has been widely described (5–7). A fine “Yin-Yang” occupancy competition mechanism between *O*-GlcNAcylation and phosphorylation on the same or adjacent Ser/Thr residues regulates protein’s interaction, stability, subcellular localization, and enzymatic activity of their common target proteins (8). Elevated nutrients uptake and metabolism are correlated with increased HBP flow, UDP-GlcNAc level, and global protein *O*-GlcNAcylation in a wide variety of cancers including chronic lymphocytic leukemia (CLL), breast, lung, liver, prostate, endometrium, pancreas, colon, larynx and bladder (9–17). This aberrant hyper-*O*-GlcNAcylation is also the result of an alteration in the expression and activity of HBP enzymes and OGT (18–21). Interestingly, decreased OGA and/or increased OGT and *O*-GlcNAcylation levels are associated with poor cancer grade progression (10, 14, 16, 20, 22, 23).

For the past two decades, a growing body of evidence has demonstrated the crucial role of abnormal *O*-GlcNAcylation of many oncogenes, tumor suppressors, and signaling actors on the growth, adhesion, migration, and invasion of cancer cells. However, while the role of *O*-GlcNAcylation in carcinogenesis and tumor progression remains of high interest in cancer research (24, 25), the impact of this glycosylation in the response of cancer to therapies is poorly investigated. The donor substrate for *O*-GlcNAcylation UDP-GlcNAc can also be epimerized to uridine diphosphate N-acetylgalactosamine (UDP-GalNAc) or modified into cytidine monophosphate-N-acetylneuraminic acid (CMP-Neu5Ac). UDP-GlcNAc, UDP-GalNAc, and CMP-Neu5Ac are nucleotide-sugar substrates for the complex glycosylation of membrane or secretory proteins. In addition to their role in the development and progression of cancer, complex glycosylation alterations have also been correlated with resistance to anti-cancer therapies by interfering with metabolism and modulating tumor cell aggressiveness (26, 27).

In this review, we summarize recent evidence highlighting that cancer therapeutics affect cellular *O*-GlcNAcylation homeostasis and, reciprocally, that *O*-GlcNAcylation modulates the response of cancer cells to therapies. Finally, we discuss the benefits of targeting *O*-GlcNAcylation as a novel promising therapeutic strategy for cancer.

## Impact of anti-cancer therapies on *O*-GlcNAcylation levels

Multiple lines of proof demonstrate that cellular stress (including glucose deprivation, chemotherapeutic and DNA-damaging agents) dramatically increases global *O*-GlcNAcylation of protein and that the sugar might be protective (28–30).

Notably, chemotherapeutic drugs (i.e. doxorubicin (DOX), 5-fluorouracil (5-FU), camptothecin (CPT), and cisplatin induce an accumulation of intracellular UDP-GlcNAc levels and global protein *O*-GlcNAcylation in different cancer cell lines (30–33). The increased flux through the HBP is at least partially mediated by the induction of Glutamine : Fructose-6-phosphate Amidotransferase (GFAT) through the AKT/X-Box Binding Protein 1 (XBP1) transcription factor pathway in an unfolded protein response (UPR)-independent manner (30) and by activation of the direct transcriptional activator Forkhead box A2 (FOXA2) (31). Interestingly, cellular UDP-GlcNAc levels are increased in cisplatin-sensitive brain tumor cells (32). Wang et al. (2021) demonstrated that cisplatin enhances UDP-GlcNAc production and global *O*-GlcNAcylation levels *in vitro* and *in vivo* by OGT, GFAT1 activation, and OGA inhibition (33). Conversely, we recently demonstrated that 5-FU decreases intracellular *O*-GlcNAcylation *in vitro* by reducing OGT at both protein and transcriptional levels but also *in vivo* most likely by reducing OGT activity (34). Owing to the fact that OGT enzymatic activity is inhibited by elevated UDP, UTP, and UDP-GlcNAc (35), we suggest that 5-FU metabolites may inhibit OGT by producing fluorinated derivates or uridine compounds (36). Overall, it seems well defined that cellular *O*-GlcNAcylation is increased in response to anti-cancer therapies-induced stress but the molecular structure of some therapeutic agents could also interfere with UDP-GlcNAc metabolism and OGT activity. UDP-GlcNAc could thus be a potential candidate for monitoring patient response to some anti-cancer therapies.

*O*-GlcNAcylation is considered a DNA damage-induced PTM since OGT relocates to the sites of damaged DNA caused by several agents (*i.e.* ionizing radiation, etoposide, methyl methanesulfonate (MMS), cisplatin, mitomycin C (MMC)) and catalyzes *O*-GlcNAcylation of several proteins of the repair machinery (37). By interfering with the other post-translational modifications of histones defining the “histone code”, it is suggested that *O*-GlcNAcylation regulates chromatin compaction and gene transcription in response to stress (38). Histone *O*-GlcNAcylation and DNA condensation are concomitantly increased under heat shock-induced stress (39). Treatment with AUY922 and ganetespib HSP90 inhibitors induces *O*-GlcNAcylation of core histones (Ser^122^ of H2A, Thr^45^ of H3, and Thr^30^ of H4) in bladder carcinoma cells (40). This study outlines the association between histone PTMs and proteomic changes in response to HSP90 inhibitor treatment in bladder carcinoma cells. Bibliographic analysis of therapies’ impact on *O*-GlcNAcylation shows a steady increase in the number of papers demonstrating that global *O*-GlcNAcylation is moderately increased by chemotherapeutic agents in sensitive cells but more significantly in resistant ones. To have a more precise vision, we will detail below the link between *O*-GlcNAcylation and anticancer response by type of therapy.

## Impact of *O*-GlcNAcylation on response to anti-cancer therapies

### 1 Targeted therapies

#### 1.1 Tamoxifen

Breast cancer is the most common cancer in women and the leading cause of cancer-related death. Among different subtypes, luminal breast cancers expressing the Estrogen Receptor alpha (ERα) represent approximately 80% of cases (41). ERα plays a crucial role in cancer initiation and progression by binding to estrogen response elements sequence in promotor of target genes upon association with estradiol, its natural ligand, and other transcription factors and activation of downstream signaling pathways such as Phosphatidylinositol 3-Kinase (PI3K)/AKT and Mitogen-Activated Protein Kinase (MAPK) (42). Hormone therapies are the mainstay of the treatment of hormone receptor positive breast tumors. Since its approval by the U.S. Food and Drug Administration (FDA) in 1977, tamoxifen is one of the most commonly used hormone therapy and acts as a partial antagonist of ERα. However, many breast tumors exhibit *de novo* or acquired resistance to hormone therapies and some potential mechanisms include deregulation of *endoplasmic reticulum* (*ER*) pathway components, modification of cell cycle, and survival regulators or activation of escape signaling pathways (43). Thus, low expression of ERα is generally associated with resistance and poor prognosis (44).

**Figure 1:**
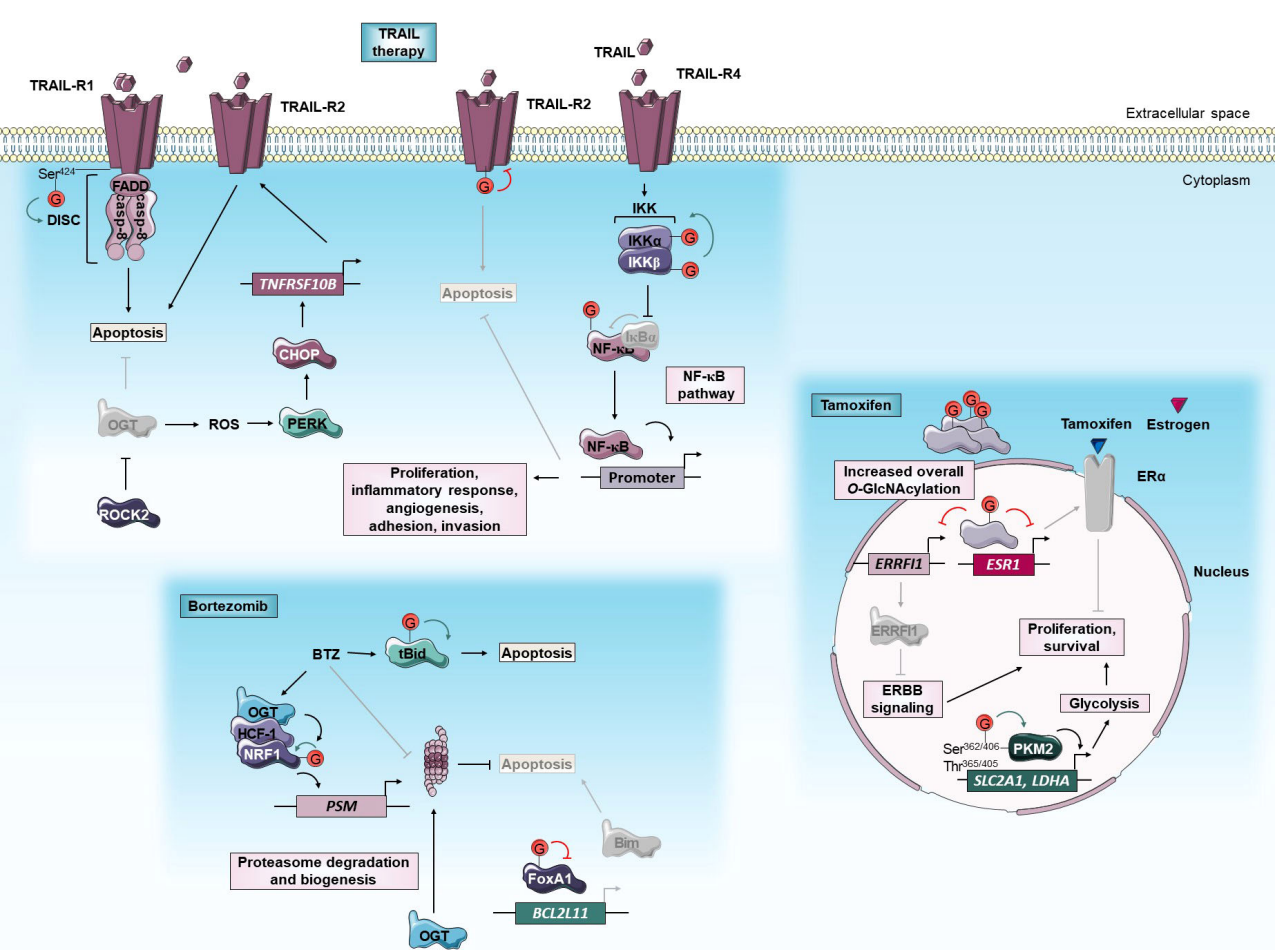
*O*-GlcNAcylation and anti-cancer targeted therapies. *O*-GlcNAcylation modulates sensitivity of cancer cells to TRAIL, bortezomib and tamoxifen targeted therapies. Green and red arrows indicate respectively activation and inhibition of protein targeted by *O*-GlcNAcylation. The gray color indicates that the protein is inactivated/absent or that the cellular mechanism is not taking place.*BCL2L11*, *Bcl-2-Like protein 11*; BTZ, bortezomib; Casp, caspase; CHOP, C/EBP Homologous Protein; DISC, death-inducing signaling complex; ERα, Estrogen receptor α; ERRFI1, ERBB Receptor Feedback Inhibitor 1; *ESR1*, *Estrogen Receptor 1*; FADD, Fas-Associated protein with Death Domain; FoxA1, Forkhead Box A1; HCF-1, Host Cell Factor-1; IKK, Inhibitory κB Kinase; *LDHA*, *Lactate Dehydrogenase A*; NF-κB, Nuclear Factor-Kappa B; NRF1, Nuclear Respiratory Factor 1; OGT, *O*-GlcNAc Transferase; PERK, Protein kinase RNA-like Kinase; PKM2, Pyruvate Kinase M2; *PSM*, *Proteasome subunit*; ROCK2, Rho-associated Coiled-coil forming protein Kinase 2; ROS, reactive oxygen species; Ser, serine; *SLC2A1*, *Solute Carrier family 2 member 1*; tBid, truncated BH3 interacting domain death agonist; *TNFRSF10B*, *Tumor Necrosis Factor (TNF) Receptor Superfamily member 10B*; Thr, threonine; TRAIL, TNF-Related Apoptosis Inducing Ligand; TRAIL-R, TRAIL Receptor.

Since cancer initiation and progression are fueled by metabolic reprogramming, the detection of metabolic biomarkers is an emerging approach for the prediction of cancer recurrence (45). In a retrospective study, Kuo et al. (2021) recently revealed that *O*-GlcNAcylation and Pyruvate Kinase M2 (PKM2) served as potentially independent prognostic markers in luminal breast cancers treated with endocrine treatment including tamoxifen. High levels of *O*-GlcNAcylation or PKM2 are positively associated with a high risk of cancer recurrence and poor long-term disease-free survival (46). PKM2 is a PK isoform preferentially expressed in cancer (47) for which hyper-*O*-GlcNAcylation is observed in breast tumors. *O*-GlcNAcylation of Ser^362^, Thr^365^, Thr^405^, and Ser^406^ causes nuclear translocation of PKM2 leading to up-regulation of GLUT1 and Lactate Dehydrogenase A (LDHA) glycolysis components. Thus, the glycosylation by targeting PKM2 would promote the Warburg effect and breast tumor growth (21, 48) (**Figure 1**).

Beyond being a recurrence biomarker of endocrine-treated breast tumors, *O*-GlcNAcylation could also be an interesting therapeutic target to sensitize anti-estrogen-resistant breast tumors. First, *O*-GlcNAcylation promotes resistance of MCF-7 HR^+^ breast cancer cells to tamoxifen by reducing ERα mRNA levels. In these cells, inhibition of OGT by siRNA or OSMI-1 specific inhibitor potentiates the cytotoxic effect of the drug (49, 50). Interestingly, tamoxifen-resistant cells are also dependent on high OGT activity. The OGT inhibition by OSMI-1 treatment sensitizes tamoxifen-resistant MCF-7 more than parental ones by inducing *ERBB Receptor Feedback Inhibitor 1* (*ERRFI1*) expression. ERRFI1 is a tumor-suppressor that inhibits ErbB receptor tyrosine kinase-signaling which is a known driver of tamoxifen resistance (51) (**Figure 1**). Thus, high expression of *ERRFI1* is associated with extended survival in patients with tamoxifen-treated ERα-positive breast tumors (52). Altogether, therapeutic approach leading to *O*-GlcNAcylation inhibition could improve the sensitivity of ERα^+^ breast cancer to tamoxifen.

#### 1.2 Bortezomib

The proteasome is a large intracellular protease complex composed of a 20S proteolytic core and two 19S regulatory particles. Proteasome activity is essential for the control of various cellular processes such as cell cycle, DNA repair, signal transduction, and protein quality control. Bortezomib (BTZ) is a peptide boronic acid that reversibly acts on the chymotrypsin-like activity of the 20S particle. BTZ has become a target of choice for the treatment of cancers that present high proteasome activity (53). Since its FDA authorization in 2003, it is used for the treatment of relapsed/refractory mantle cell lymphoma (MCL) and multiple myeloma and is further undergoing clinical evaluation in solid tumors including breast, colorectal, ovarian, pancreatic, prostate, and squamous cell carcinomas. However, innate and acquired resistance to BTZ are frequently observed. Some BTZ resistance mechanisms include mutations or up-regulation of proteasome subunits, alteration of stress response, and cell survival pathways, or multi-drug resistance (54).

Interestingly, OGT is included in the list of BTZ sensitizers in myeloma cells (55). Several pieces of evidence demonstrate that *O*-GlcNAcylation up-regulates the bounce-back response that restores proteasome activity by transcriptional activation of proteasome subunit genes. There is a positive correlation between OGT and proteasome subunits expression in clinical cancer samples including breast invasive carcinoma and colorectal adenocarcinoma. Sekine et al. (2018) firstly demonstrated that *O*-GlcNAcylation is critical for the maintenance of proteasome activity by regulating Nuclear Respiratory Factor 1 (NRF1) transcription factor through its interaction with Host Cell Factor-1 (HCF-1). In response to BTZ, OGT targets and stabilizes NRF1. In turn, HCF-1 promotes the binding of NRF1 at promoter regions of proteasome subunit genes. *OGT* knock-down sensitizes MDA-MB-231 breast and NCI-460 pancreatic cancer cells to BTZ *in vitro* and in xenograft in a mouse model by blocking NRF1-dependent proteasome bounce-back response (56). It was also demonstrated that *O*-GlcNAcylation promotes the bounce-back response in an NRF1-independent-manner by promoting the turnover of the proteasome. Under BTZ-induced proteasome inhibition, *O*-GlcNAcylation enhances both degradation and biogenesis of proteasome allowing the recovery of its activity (**Figure 1**). The underlying mechanism is not yet clarified but the translation or the stability of proteasome subunits may be improved by *O*-GlcNAcylation since neither the subunit mRNA levels of the proteasome nor its assembly pathway is affected by OGT inhibition (57).

Other studies reveal that *O*-GlcNAcylation could regulate the BTZ-mediated activation of extrinsic and intrinsic apoptosis triggered by the accumulation of pro-apoptotic proteins including BH3 interacting domain death agonist (Bid) and Bim proteins (58). Increased *O*-GlcNAcylation of FOXA1, a forkhead transcription factor that activates Bim expression, is involved in breast cancer resistance to BTZ. There is an association between the elevation of *O*-GlcNAcylation content and resistance to BTZ in mammary cancer samples. BTZ dynamically induces an increase of global *O*-GlcNAcylation levels in intrinsic and extrinsic BTZ resistant breast cell lines but not in sensitive ones. In BTZ resistant cells, *O*-GlcNAcylation targets and reduces the stability of FOXA1 allowing Bim attenuation. OGT silencing or inhibition with L01 small molecule sensitizes resistant cells to BTZ by increasing FOXA1 and Bim protein levels (59). In contrast, Luanpitpong et al. (2019) demonstrated that *O*-GlcNAcylation of truncated Bid (tBid) pro-apoptotic protein could sensitize MCL to BTZ. The use of pharmacological inhibitors of OGT (alloxan) and OGA (PUGNAc, Thiamet-G, ketoconazole (KCZ)) respectively abrogate and sensitize to BTZ-induced apoptosis in MCL cell lines. *O*-GlcNAcylation targets tBid and interferes with its ubiquitination and its subsequent degradation by the 26S proteasome (60). Glycosylation-mediated stabilization and accumulation of tBid intensify the apoptosis signal induced by BTZ (**Figure 1**). Thus, OGA inhibition by KCZ treatment increased the sensitivity of patient-derived primary cells and BTZ-resistant MCL cells (61).

Together, these data indicate that, in a cancer type-dependent manner, activation or inhibition of *O*-GlcNAcylation in combination with BTZ proteasome inhibitor is a promising clinical strategy against resistance to therapy.

#### 1.3 Tumor necrosis factor-related apoptosis inducing ligand therapy

Upon Tumor necrosis factor (TNF)-Related Apoptosis Inducing Ligand (TRAIL) cytokine binding, TRAIL-Receptor 1 and 2 (TRAIL-R1 and TRAIL-R2 also known as respectively Death Receptor 4 and 5 (DR4 and DR5)) trigger the assembly of death-inducing signaling complex (DISC). The latter leads to apoptosis by activation of initiator caspases-8/10 and downstream effector caspases such as caspase-3. Unlike TNF-α and FAS extrinsic apoptosis-inducing ligands, TRAIL has an attractive ability to selectively induce apoptosis in tumor cells while sparing normal cells (62). Therefore, several clinical trials are currently underway to assess the efficacy of TRAIL therapy in cancer. Circularly permuted TRAIL (CPT) is tested in myeloma and some antibodies have also been developed: an anti-TRAIL-R (dulanermin) against lymphoma, colorectal and lung cancers, an anti-TRAIL-R1 (mapatumumab) or an anti-TRAIL-R2 (anti-DR5) (tigatuzumab) against several solid tumors (63). However, such clinical trials have failed to achieve a beneficial anticancer activity because a large number of cancers develop intrinsic and acquired resistance to TRAIL. Inhibition of apoptosis can be due to impaired TRAIL-R signaling, reduced caspases function, or disrupted balance between pro-apoptotic and anti-apoptotic proteins (64).

The death domain (DD) of TRAIL-R1 regulates both apoptosis and necrosis upon TRAIL ligand binding. In response to TRAIL, *O*-GlcNAcylation at the Ser^424^ DD residue of TRAIL-R1 facilitates receptors clustering within lipid rafts, DISC assembly, and induction of cell death (**Figure 1**). Several TRAIL-resistant cancer cell lines display a TRAIL-R1-Ser^424^ mutation and therefore defect of *O*-GlcNAcylation. The mutation at Ser^424^ residue could then be a potential genetic diagnostic marker of patients with cancer to predict TRAIL therapy response (65). *O*-GlcNAcylation of TRAIL-R2 also plays an important role in pancreatic cancer TRAIL resistance (66). Gain- and loss-of-function of OGT respectively render TRAIL-sensitive pancreatic cells more resistant to tigatuzumab TRAIL-R2 agonist and promote tigatuzumab-induced apoptosis in TRAIL-resistant cells *in vitro* and in a mouse model. Since *O*-GlcNAcylation of TRAIL-R2 regulates its trimerization and the activation of apoptotic signals, inhibiting *O*-GlcNAcylation could increase the effectiveness of TRAIL therapy (**Figure 1**). In addition, the overall level of global *O*-GlcNAcylation or *O*-GlcNAcylated TRAIL-R2 could be a biomarker of pancreatic cancer sensitivity to TRAIL therapy. In addition to activating apoptosis, TRAIL has also been described to induce the Nuclear Factor-Kappa (NF-κB) survival pathway that may promote drug resistance (67). Several studies have already revealed that *O*-GlcNAcylation regulates this pathway (68–72). Recently, Lee et al. (2021) demonstrate that a combination of TRAIL and OSMI-1 (OGT inhibitor) enhances TRAIL-induced apoptosis in colon cancer cells and xenograft tumors by lowering the activity of Inhibitory κB Kinase β (IKKβ) and inhibition of downstream NF-κB pro-survival signaling (73) (**Figure 1**). Additionally, OGT inhibition by OSMI-1 improves TRAIL-induced apoptosis by a parallel mechanism involving *ER* stress response. Hypo-*O*-GlcNAcylation induces the accumulation of reactive oxygen species (ROS) that leads to *ER* stress and activation of UPR signaling pathways including Protein Kinase RNA (PKR)-like kinase (PERK). Activation of PERK enhances expression of the pro-apoptotic protein C/EBP Homologous Protein (CHOP) that, in turn, up-regulates expression of TRAIL-R2 leading to TRAIL sensitization (73) (**Figure 1**). Concordantly, *O*-GlcNAcylation prevents activation of CHOP (74). Together, these studies suggest that a combination of TRAIL and OGT inhibition may induce synergistic effects and provide a promising therapeutic strategy for the treatment of various cancers.

Finally, a recent study identifies the GTPase RhoA effector Rho-associated Coiled-coil forming protein Kinase 2 (ROCK2) as a key regulator of *O*-GlcNAcylation and TRAIL sensitivity in osteosarcoma (OS). ROCK2 inhibits TRAIL-mediated apoptosis in OS cells by affecting OGT ubiquitination and degradation, and subsequently increasing protein *O*-GlcNAcylation levels (**Figure 1**). ROCK2 overexpression is an independent predictor of poor prognosis in OS patients and knock-down of ROCK2 causes a reduction in *O*-GlcNAcylation level and increases the OS cell sensitivity to TRAIL. Based on these data, the authors suggest ROCK2 as a potential biomarker for OS diagnostic and therapeutic tools (75).

### 2 Chemotherapies

#### 2.1 5-fluorouracil

5-FU is a fluorinated uracil analog that acts as an antimetabolite to disrupt nucleic acid synthesis and repair in highly proliferating cancer cells. It was approved in 1962 by FDA and is widely used in the treatment of solid tumors including colorectal, breast, anus, esophagus, pancreas, stomach, head, neck, and ovary cancers. The major cytotoxic activity of 5-FU consists of its conversion by Thymidine Kinase (TK) and Thymidine Phosphorylase (TP) into the active metabolite 5-fluorodeoxyuridine monophosphate (FdUMP) which sequesters and inhibits Thymidylate Synthase (TS), a key enzyme involved in the *de novo* synthesis of deoxythymidine monophosphate (dTMP). Cancer resistance to 5-FU can be caused by an alteration in metabolism, TS target level, recognition or repair of DNA damages, and/or inhibition of apoptosis (76).

**Figure 2:**
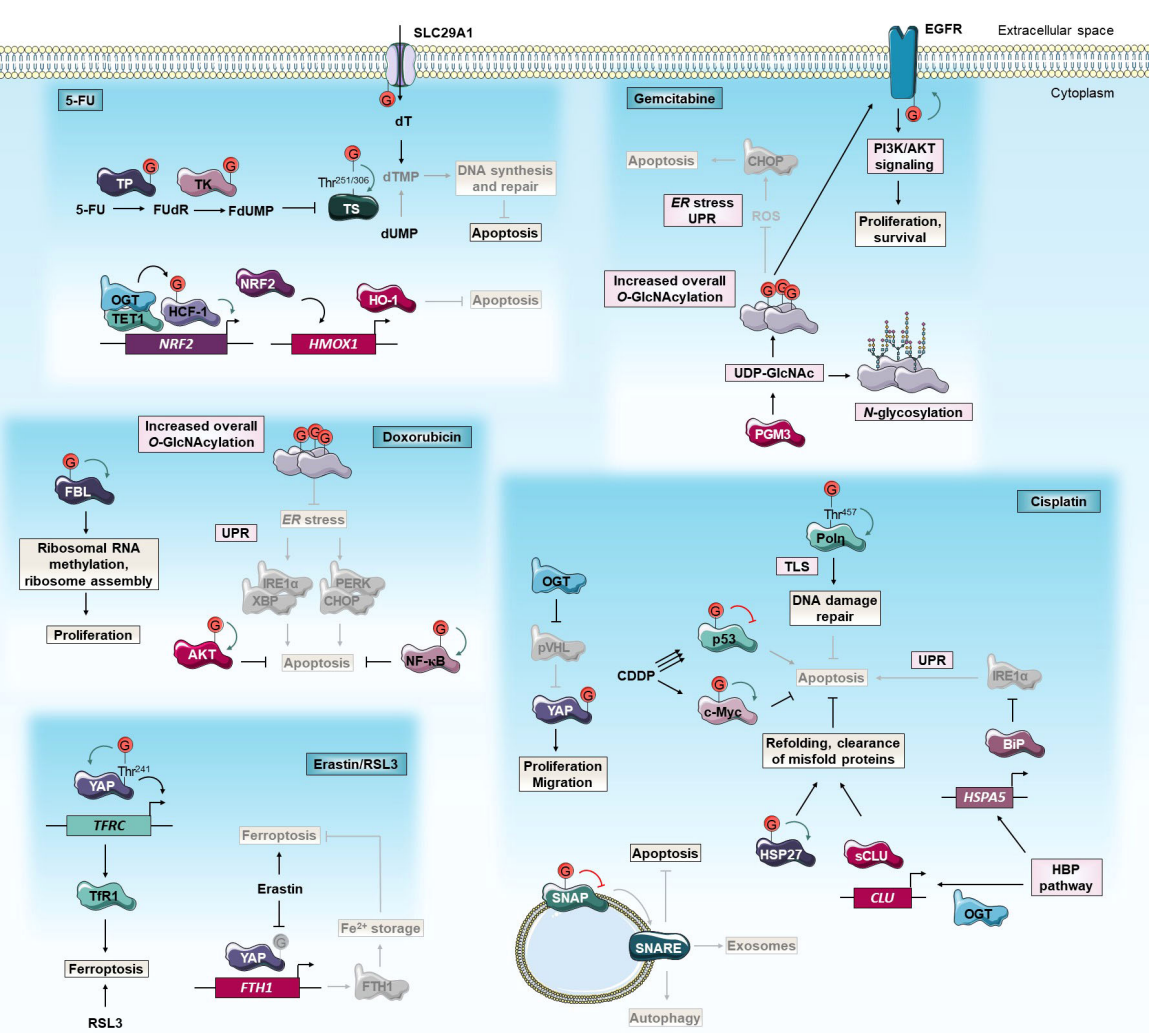
*O*-GlcNAcylation and anti-cancer chemotherapies. *O*-GlcNAcylation modulates cancer cell response to 5-FU, cisplatin, gemcitabine, doxorubicin and erastin/RSL3 chemotherapies. Green and red arrows indicate respectively activation and inhibition of protein targeted by *O*-GlcNAcylation. The gray color indicates that the protein is inactivated/absent or that the cellular mechanism is not taking place.5-FU, 5-fluorouracil; BiP, Binding immunoglobulin Protein; CDDP, cis-diaminedichloroplatinium(II); CHOP, C/EBP Homologous Protein; *CLU*, *Clusterin*; dUMP, deoxyuridine monophosphate; dT, deoxythymidine; dTMP, dT monophosphate; EGFR, Epidermal Growth Factor Receptor; ER, *endoplasmic reticulum*; FBL, Fibrillarin; *FTH1*, Ferritin Heavy chain 1; FdUMP, fluorodUMP; FUdR, fluorodeoxyuridine; HBP, Hexosamine Biosynthetic Pathway; HCF-1, Host Cell Factor-1; *HMOX1*, *Heme Oxygenase-1*; HO-1, Heme Oxygenase-1; HSP27, Heat Shock Protein 27 kD; IRE1α, Inositol-Requiring Enzyme 1α; NF-κB, Nuclear Factor-Kappa B; *NRF2*, Nuclear factor erythroid-2-Related Factor 2; OGT, *O*-GlcNAc Transferase; PERK, Protein kinase RNA-like Kinase; PGM3, Phosphoacetylglucosamine Mutase 3; PI3K, Phosphatidylinositol 3-Kinase; Polη, DNA Polymerase η; pVHL, von Hippel-Lindau protein; ROS, reactive oxygen species; RSL3, Ras-selective lethal small molecule 3; sCLU, secretory CLU; SLC29A1, Solute Carrier family 29 member 1; SNAP, Synaptosomal Associated Protein; SNARE, Soluble N-ethylmaleimide-sensitive-factor Attachment protein Receptor; TET1, Tet methylcytosine dioxygenase 1; TfR1, Transferrin Receptor 1; *TFRC*, Transferrin Receptor; Thr, threonine; TK, Thymidine Kinase; TLS, translesion DNA synthesis; TP, Thymidine Phosphorylase; TS, Thymidylate Synthase; UDP-GlcNAc, uridine disphosphate N-acetylglucosamine; UPR, unfolded protein response; XBP, AKT/X-Box Binding Protein; YAP, Yes-Associated Protein.

Several studies have already correlated *OGT* expression with 5-FU sensitivity. Notably, Temmink et al. (2010) showed that H630 colon cancer cells resistant to trifluorothymidine (TFT), a fluorinated thymidine analog that is part of TAS-102 chemotherapy and shares the anabolic pathway of TS inhibition with 5-FU, underexpress OGT, Solute Carrier family 29A (SLC29A), and TK (77). On the contrary, a transcriptomic study on human NCI-60 tumors showed that OGT expression is negatively correlated with FdUMP sensitivity, no correlation with 5-FU sensitivity was established (78). OGT was also identified in a cluster of co-expressed genes associated with 5-FU resistance in colorectal cancer (CRC) (79). Moreover, the 5-FU pathway actors SLC29A1 (80), TP (10), TK (81, 82), and TS (34) are direct targets for *O*-GlcNAcylation (**Figure 2**). Nevertheless, modified sites and roles of this PTM on these proteins as well as their impacts on 5-FU sensitivity are largely not elucidated yet. Very recently, we demonstrated that TS is stabilized by *O*-GlcNAcylation at Thr^251^ and Thr^306^ residues. *OGT* knock-down decreases 5-FU-induced apoptosis by enhancing TS proteasomal degradation and, by reducing TS level and enzyme activity in HT-29 cell line but not in 5-FU resistant HT-29 counterpart (34) (**Figure 2**). The latter exhibits *TS* gene amplification and TS overexpression which is a current biomarker of 5-FU resistance in CRC (83). Our data highlight the importance to distinguish *TS* gene overexpression and the corresponding enzyme post-translational stabilization. We have shown that the regulation of 5-FU response by *O*-GlcNAcylation is finely tuned and depends on a proper quantity of TS proteins. In a CRC mouse model, the combination of 5-FU with the OGA inhibitor Thiamet-G had a synergistic inhibitory effect on tumor grade and progression (34). As the *O*-GlcNAcylation homeostasis-TS axis mediates the response to 5-FU, we propose to combine an OGA inhibitor with 5-FU-based therapies to enhance CRC patient response to 5-FU.

Another study showed that, in comparison to parental cells, 5-FU resistant SNUC5 colon cancer cells undergo oxidative stress due to 5-FU-induced accumulation of ROS. These cells overexpressed Tet methylcytosine dioxygenase 1 (TET1) and OGT. Both enzymes interact together at the promoter of Nuclear factor erythroid-2-Related Factor 2 (NRF2), a transcription factor that regulates the expression of antioxidant enzymes such as Heme Oxygenase-1 (HO-1). OGT indirectly activates the transcription of *NRF2* (**Figure 2**). Since oxaliplatin-resistant SNUC5 cells and cisplatin-resistant ovarian cancer cells express a high level of NRF2, inhibition of *O*-GlcNAcylation may potentiate the 5-FU-induced oxidative stress in these cells by decreasing NRF2 and HO-1 expression (84, 85). Chen et al. (2017) revealed an opposite functional connection between *O*-GlcNAcylation and NRF2 antioxidant pathway. In fact, Kelch-like ECH-Associated Protein 1 (KEAP1), the primary negative regulator of NRF2, *O*-GlcNAcylation at Ser^104^ is required for proteasomal degradation of NRF2. Gene expression signatures of low OGT activity and high NRF2 activation are strongly correlated in several breast tumor datasets and OGT inhibition induces NRF2 and subsequently reduces cysteine-deprivation induced-oxidative stress in breast MDA-MB-231 cells (86). Additionally, the inhibitory effect of *O*-GlcNAcylation on oxidative stress was confirmed as GFAT inhibition with 6-diazo-5-oxo-norleucine (DON) sensitizes cancer cells to oxidative stress-induced apoptosis (83). Of note, oxidative stress-resistant Phosphatase and tensin homolog (Pten) KO-mice tumors show increased GFAT2 and *O*-GlcNAcylation levels compared to wild-type (87).

#### 2.2 Cisplatin

Cisplatin is a platinum-based alkylating agent that, mechanistically, reacts with DNA bases to cross-link adjacent purines, blocks DNA replication and induce apoptosis in rapidly dividing cells. It was firstly approved by FDA in 1978 for the treatment of testicular, ovarian, and bladder cancers. Nowadays, it is an important component of combination therapy for a wide range of solid tumors. The resistance to cisplatin can be caused by decreased drug accumulation, increased drug detoxification, increased DNA repair or DNA damage tolerance, and cell death escape (68). We describe below how *O*-GlcNAcylation interferes with the three last resistance mechanisms.

Autophagy is a self-protection mechanism that occurs as an emergency response and consists of phagocytosis of cytoplasmic proteins or damaged organelles into autophagosomes to meet the basic metabolic needs of cells. This cellular defensive pathway has a pro-survival role but can induce cell death under certain conditions. Autophagy plays a crucial role in the response of cancer cells to chemotherapeutic drugs including cisplatin (69, 70). Zhou et al. (2018) showed that OGT expression is significantly lower in cisplatin chemoresistant ovarian cancerous tissues compared to sensitive tissues. Additionally, *OGT* knock-down increases resistance to cisplatin of ovarian cancer cells and xenografted in mice (71). Reduced OGT expression regulates Synaptosomal Associated Protein 29 (SNAP 29) component of the Soluble N-ethylmaleimide-sensitive-factor Attachment protein Receptor (SNARE) vesicle formation machinery (72) which increases pro-survival autophagic flux that correlates with decreased apoptosis and increased cisplatin resistance (71) (**Figure 2**). Conversely, Wang et al. (2018) showed that siRNA invalidation of OGT increases autophagic flux and cisplatin-induced apoptosis in T24 and UMUC-3 bladder cancer cells (17, 88) (**Figure 2**). In addition to the degradation of long-lived macromolecules, the autophagy-lysosome system has broader functions and is involved in the selective degradation of other intracellular components including some oncogenes. The transcriptional co-activator Yes-Associated Protein (YAP) is a major effector of the Hippo pathway and its overactivation promotes cancer cell growth and chemoresistance. *O*-GlcNAcylation can block the von Hippel-Lindau protein (pVHL)-mediated lysosomal degradation of YAP and thus enhance cellular proliferation, migration, and cisplatin resistance in lung adenocarcinoma cells (89) (**Figure 2**). SNARE proteins also regulate the fusion of exosome-containing multivesicular bodies (MVB) to the plasma membrane and their secretion into the extracellular environment. Compared to normal cells, cancer cells secrete an increasing number of exosomes that can facilitate the efflux of intracellular chemotherapeutic drugs, hence promoting chemoresistance (90). In A2780 and SKOV3 ovarian cancer cells, down-regulation of OGT reduces *O*-GlcNAcylation of SNAP-23 promoting the formation of SNARE complex and exosomes. Thus, this *O*-GlcNAcylation-mediated mechanism causes chemoresistance by increasing the exosome-mediated efflux of intracellular cisplatin (91) (**Figure 2**).

The tumor suppressor p53 plays a crucial role in cell biology and is considered the “guardian of the genome”. In response to DNA damages, caused by chemotherapeutic agents such as cisplatin, p53 is stabilized and activates downstream pathways to arrest the cell cycle, repair the damaged DNA or induce apoptosis. Loss of function of p53 is a common feature in more than 50% of human cancers (92) and drives tumor growth and chemotherapy resistance. Several studies reveal that *O*-GlcNAcylation could regulate cisplatin resistance in a p53-dependent manner. Luanpitpong et al. (2017) showed that, depending on the cellular context and the level of cisplatin-activation of p53, increased *O*-GlcNAcylation could lead to cisplatin resistance in lung cancer cells by distinct molecular mechanisms. Indeed, in NCI-H292 cells, cisplatin weakly induces p53 activation and *O*-GlcNAcylation stabilizes the c-Myc oncoprotein to promote cell survival. In parallel, in NCI-H460 cells in which cisplatin induces strong activation of p53, *O*-GlcNAcylation of p53 increases its ubiquitination and its proteasomal degradation resulting in an enhanced oncogenic and anti-apoptotic phenotype (19) (**Figure 2**). Of note, literature shows some discrepancy since previous studies showed that *O*-GlcNAcylation stabilizes p53 (93) as in ovarian cancer cells (94). In these cells, the combination of OGA inhibitor Thiamet-G with cisplatin increases the drug sensitivity by inducing cell cycle arrest in wild-type p53 and to a lesser extent in silenced p53 cells. These data suggest that the Thiamet-G and cisplatin synergistic anti-tumoral effect is partially dependent on wild-type p53 pathway activation but also on other unknown growth-related pathways (94). Altogether, these results indicate that the effect of *O*-GlcNAcylation on cisplatin chemotherapy response could depend on the type of cancer and/or induction level, mutation status, and site-specific *O*-GlcNAcylation of p53.

After cisplatin exposure, DNA damage responses are initiated to maintain genome integrity. DNA cross-links are removed by the Nucleotide Excision Repair (NER) pathway or bypassed during replication through translesion DNA synthesis (TLS). TLS is mediated by specialized DNA polymerases (Pol) including Polη characterized by a low fidelity and the ability to replicate damaged DNA on stalled replication forks. TLS process is believed to contribute to the development of cisplatin resistance (95). In response to DNA damage, OGT relocates to DNA lesions and catalyzed *O*-GlcNAcylation of several substrates (37). Notably, Polη undergoes *O*-GlcNAcylation at Thr^457^ which allows its polyubiquitination at Lys^462^ and its subsequent dissociation from replication forks. Thus, cells expressing the *O*-GlcNAc-deficient T457A mutant Polη exhibit a higher sensitivity to cisplatin (96) (**Figure 2**). Given the key role of Polη in TLS and cisplatin chemoresistance of ovarian cancer stem cells (95), targeting Polη *O*-GlcNAcylation could be a chemotherapy-enhancing strategy in ovarian cancer treatment.

Stress conditions can destabilize the folding of cytoplasmic proteins leading to exposure of hydrophobic regions that interact with each other to form deleterious proteolysis resistant aggregates. In these conditions, molecular chaperones can form multimeric complexes to refold denatured or aggregated proteins. Elevated activity of chaperones allows cancer cells to grow and resist stress conditions including chemotherapy (97). Several studies showed that *O*-GlcNAcylation modulates cisplatin response by targeting directly or indirectly molecular chaperons. Based on the initial observation that higher *O*-GlcNAcylation levels in hepatoblastoma tissues might be associated with the pathogenesis, Song and collaborators (2019) realized a global proteomic analysis using *O*-GlcNAc antibody beads and immobilized metal affinity chromatography (IMAC) enrichments followed by liquid chromatography coupled to tandem mass spectrometry (LC-MS/MS) to identify potential *O*-GlcNAcylated and phosphorylated therapeutic targets. Among them, the anti-apoptotic chaperone Heat Shock Protein 27 kD (HSP27, also known as Heat Shock Protein family Beta-1 (HSPB1)) was identified, and its *O*-GlcNAcylation was further shown to promote cell proliferation and cisplatin resistance in hepatic cancer cell lines (98) (**Figure 2**). Overexpression of HSP27 is related to tumorigenesis, metastasis, and therapy resistance by acting as an upstream regulator of oncogenic (*i.e.* Hippo pathway, AKT, Glycogen Synthase Kinase 3 Beta (GSK3β), or β-catenin) and anti-apoptotic (i.e. NF-κB, Mothers Against Decapentaplegic homolog 3 (SMAD3), p38/MAPK) pathways. Targeting HSPs to enhance the effects of anti-cancer drugs is a promising approach and several molecules that modulate HSP protein functions are currently investigated in preclinical and clinical trials. For instance, HSP70 client protein inhibitor sorafenib has been approved to treat hepatocellular, renal, and thyroid carcinoma (97, 99). Studies revealed that *O-*GlcNAcylation stimulates aggressiveness and resistance of several cancers to chemotherapies and targeted therapies by modulating levels, activity, or subcellular localization of HSP proteins (100) including HSP27 (101–103). In this way, decreasing *O*-GlcNAcylation of HSPs could be a new anti-cancer therapeutic strategy. Secretory clusterin (sCLU) is a chaperon that facilitates the extracellular clearance of misfolded proteins. Like HSP proteins, this pro-survival factor is involved in cancer cell proliferation and drug resistance. OGT and sCLU expression are elevated in cervical cancer cell lines, and *O*-GlcNAc-induced up-regulation of sCLU leads to cisplatin resistance (**Figure 2**). sCLU silencing antisense oligonucleotides have been developed and approved for anti-cancer clinical trials. However, there is no drug currently available that completely blocks sCLU expression. Then, inhibiting OGT in combination with the knock-down of sCLU could be a new therapeutic strategy to overcome cisplatin resistance (104). Binding immunoglobulin Protein (BiP) is a major *ER* HSP70 family chaperone that binds to misfolded or unfolded proteins and releases UPR sensors (Inositol-Requiring Enzyme 1α (IRE1α), PERK, and Activating Transcription Factor 6 (ATF6)) to initiate the UPR pathway. Inhibition of the HBP pathway with the glutamine analog DON enhances cisplatin-induced apoptosis of lung cancer cells by repressing BiP expression and activating IRE1α (**Figure 2**). Up-regulation of BiP expression by HBP flux is supported by a previous study carried out in hepatoblastoma cells (105). Together, these data suggest that the combination of cisplatin with HBP inhibitors could improve lung cancer platinum-based chemotherapies (106).

#### 2.3 Gemcitabine

Gemcitabine (GEM) acts as an antimetabolite and is a deoxycytidine nucleoside analog. It was approved by FDA in 1996 as chemotherapy for pancreatic cancer and later on for non-small cell lung, ovarian and breast cancers. GEM is metabolized to the active metabolite, 2’,2’-difluoro-2’-deoxycytidine triphosphate (dFdCTP) that can incorporate into DNA and RNA leading to the termination of DNA synthesis and faulty translation respectively. However, many cancers resist to GEM and the main involved mechanism consists of an up-regulation of the catabolic enzyme Cytidine Deaminase (CDA), deficiency in the anabolism enzyme Deoxycytidine Kinase (DCK), and alterations in nucleoside influx transporters (107).

Interestingly, increased metabolism of glucose and glutamine, two substrates for the HBP, fuel GEM resistance of pancreatic cancer (108, 109). Moreover, the HBP enzyme Phosphoacetylglucosamine Mutase 3 (PGM3) was shown to be overexpressed in GEM resistant human pancreatic tumors. Upon increasing GEM doses, PGM3 and global protein *O*-GlcNAcylation levels are decreased in sensitive BxPC-3 pancreatic cancer cells but increased in PANC-1 and MIA PaCa-2 resistant ones (110). Based on these observations, Ricchiardiello et al. (2020) recently investigated the effect of HBP flux inhibition in avoiding GEM resistance. Treatment with PGM3 inhibitor FR054 decreases tri-/tetra-antennary complex N-glycosylation of membrane proteins, *O*-GlcNAcylation of intracellular proteins, and enhances GEM efficiency *in vitro* and *in vivo*. Mechanistically, chemical inhibition of PGM3 causes a sustained activation of the pro-apoptotic UPR protein CHOP associated with a reduction in membrane localization of pro-tumorigenic Epidermal Growth Factor Receptor (EGFR) and a strong attenuation of the EGFR-Akt axis (110) (**Figure 2**). It was recently demonstrated that EGFR is directly *O*-GlcNAcylated and that *OGT* knock-down downregulates EGFR and its downstream PI3K/AKT pathway, and promotes cell cycle arrest and apoptosis in 786-O renal cell carcinoma cells (111). Pending further investigations to determine whether hypo-*O*-GlcNAcylation induced by PGM3 inhibition is directly involved in GEM sensitization mechanism, we speculate that hypo-*O*-GlcNAcylation could activate CHOP by inducing ROS accumulation, *ER* stress, and UPR activation (73, 74) (**Figure 2**). Therapeutic approaches involving PGM3 inhibition, and possibly inhibition of protein *O*-GlcNAcylation, in combination with GEM could be promising to bypass the drug resistance in pancreatic cancer.

#### 2.4 Doxorubicin

Doxorubicin (DOX) is an anthracycline class medication approved by FDA and routinely applied to the treatment of several cancers such as breast, lung, gastric, ovarian, thyroid, non-Hodgkin’s, and Hodgkin’s lymphoma, sarcoma and pediatric tumors. This chemotherapeutic agent acts by intercalation with DNA, disruption of Topoisomerase II (Top-II), and production of quinone-type free radicals triggering cell death. Chemoresistance involves alteration of efflux ATP-Binding Cassette (ABC) transporter proteins, epigenetics, and signaling pathways (e.g. MAPK, PI3K/AKT/mammalian Target Of Rapamycin (mTOR), Wnt/β-catenin, Notch and Transforming Growth Factor Beta (TGF-β)) (112).

As previously mentioned, DOX treatment stimulates HBP flux and protein *O*-GlcNAcylation through the XBP1/AKT axis and FOXA2 leading to activation of pro-survival pathways (30, 31). Interestingly, recent studies showed that DOX treatment in combination with OSMI-1 has a synergistic apoptotic effect not only in several cancer types including sensitive and resistant liver (30, 113), prostate (114), and breast cancer cell lines but also in primary cells from newly diagnosed, refractory and relapsed acute myeloid leukemia (AML) patients (30). Hypo-*O*-GlcNAcylation enhances the sensitivity toward DOX by preventing pro-survival NF-κB and AKT activation (30, 113) and promoting *ER* stress response which leads to increased IRE1α-XBP and PERK-CHOP signaling (113) (**Figure 2**). Conversely, *O*-GlcNAcylation could protect MCF-7 breast cancer cells against DOX by stabilizing box C/D small nucleolar ribonucleoprotein complexes (snoRNPs) core component fibrillarin (FBL) on Ser^142^ and maintaining ribosomal RNA methylation and ribosome assembly (110) (**Figure 2**). Dysregulation of ribosome biogenesis promotes cancer cell proliferation (115). To note that treatment of HepG2 hepatocarcinoma cells with a low dose of DOX (1 μM) or a suboptimal dose of OSMI-1 (20 μM) alone did not induce apoptosis while the combined treatment did (113). In addition to improve therapeutic efficacy and alleviate DOX resistance in different cancer types, therapy combination with OGT inhibition could allow the use of smaller doses of DOX, hence reducing the associated side effects.

#### 2.5 Erastin/RSL3

Ferroptosis is a newly identified form of programmed cell death that is characterized by an iron-dependent accumulation of lipid peroxides. Induction of ferroptosis emerged as a new strategy to trigger cancer cell death and ferroptosis-inducing compounds have been categorized into two classes based on their inhibition mode of Glutathione Peroxidase 4 (GPX4). The latter detoxifies lipid hyperoxides within biological membranes. The first class (e.g., erastin, Sorafenib) indirectly inhibits GPX4 by blocking the cysteine-glutamate transporter (system X_C_^-^) and depleting the GPX4 cofactor glutathione (GSH). The second class (e.g., Ras-selective lethal small molecule 3 (RSL3)) directly inhibits GPX4 leading to lipid-ROS accumulation (116). YAP has been reported to play a pivotal role in ferroptosis by upregulating critical modulators including Acyl-CoA Synthetase Long-chain family member 4 (ACSL4), Transferrin Receptor 1 (TfR1) (117), and Ferritin heavy chain 1 (FTH1) (118). *O*-GlcNAcylation of YAP on Thr^241^ residue prevents its degradation and enhances its transcriptional activity (12, 119). Under ferroptotic stress with erastin, system X_C_^-^ inhibition leads indirectly to Thr^241^ hypo-*O*-GlcNAcylation of YAP, FTH1 down-regulation, free iron release, and increased ferroptosis in lung adenocarcinoma cells (118) (**Figure 2**). In contrast, Zhu et al. (2021) demonstrate that, under high glucose levels, *O*-GlcNAcylation enhances RSL3-mediated ferroptosis by targeting YAP on Thr^241^ and increasing its transcriptional activity on TfR1 in HCC cell lines (120) (**Figure 2**). Further study of YAP *O*-GlcNAcylation’s role in ferroptosis, which seems to depend on the ferroptosis-inducing compound and the type of cancer tissue, would be useful to clarify these discrepancies.

### 3 Immunotherapy

Programmed cell Death protein 1 (PD-1) is a suppressive receptor expressed in immune T cells upon T cell activation. It is involved in T cell activation, proliferation, survival, and cytotoxic activity. PD-1 is highly selective for Programmed Death-Ligand 1 (PD-L1), an immune-inhibitory ligand overexpressed by cancer cells as an “adaptive immune mechanism” to escape immune responses. A range of monoclonal antibodies specific to the PD-1 and PD-L1 immune checkpoints have been approved for the treatment of a wide variety of human cancers. However, compensatory up-regulation of PD-L1 gradually causes drug resistance in some cancer patients (121).

Shang et al. (2021) recently showed that the embryo- and tumor-specific folate cycle enzyme Methylenetetrahydrofolate Dehydrogenase 2 (MTHFD2) can rescue cancer cells from T cell cytotoxicity by driving the folate cycle and by sustaining sufficient uridine-related metabolites including UDP-GlcNAc substrate donor. Thus, MTHFD2 is highly positively correlated to global *O*-GlcNAcylation levels which, in turn, targets c-Myc at Thr^58^ and promotes its stability and its direct activation of PD-L1 transcription (122). The c-Myc *O*-GlcNAcylation at Thr^58^ prevents its interaction with F-box/wD repeat-containing protein 7 (Fbw7) ubiquitin E3-ligase, increases its stability, and its transcriptional activity on target genes involved in cell proliferation, metabolism, and apoptosis (123). More particularly, *O*-GlcNAcylation mediated-stabilization of c-Myc is involved in renewal, clonal expansion and malignant transformation of T cell progenitors (88). MTHFD2, which is rarely expressed in normal adult tissues, emerges as a potential safe target. Thus, inhibiting the *O*-GlcNAcylation-c-Myc-PD-L1 signaling axis could be a promising therapeutic strategy to stimulate anti-cancer T cell cytotoxicity, hence improving immunotherapy.

### 4 Radiotherapy

Radiation therapy is given to about 50% of all cancer patients and contributes to 40% of curative anti-cancer treatments. The mode of action is to prevent cancer cells from proliferation by inducing extensive DNA damage. Exposure to radiation leads to DNA double-strand breaks (DSB) and triggers the DNA damage response (DDR), a complex signal transduction pathway that regulates DNA repair, cell cycle checkpoints activation, chromatin remodeling, cell senescence, and apoptosis. One of the key DNA damage-induced epigenetic modifications is the phosphorylation of H2A histone family member X (H2AX, also known as γH2AX) by a group of PI3-like kinases including Ataxia Telangiectasia Mutated (ATM), ATM and Rad3 related protein (ATR) and DNA-dependent Protein Kinase (DNA-PK). Aberrant DDR pathway activation directly confers tumor radioresistance (124).

In non-irradiated cells, OGT functions to suppress genomic instability and reduce cellular stress probably by protecting cells from oxidative stress and/or cell-cycle defects. Thus, under oxidative stress conditions, increased OGT and *O*-GlcNAc levels were required to induce DDR and proliferation of *Drosophila* intestinal stem/progenitor cells. In an autoregulatory feedback loop, Checkpoint Kinase 1 (CHK1)/CHK2 DDR effectors stabilize OGT (125). Moreover, OGT inhibition can induce cellular stress resulting in constitutive activation of DDR (126). *O*-GlcNAcylation can directly target many proteins of the DDR in response to heat-stress-induced DNA damage (*i.e.* DNA-PK, Coactivator-associated Arginine Methyltransferase 1 (CARM1), Ubiquitin-Associated Protein 2-Like (UBAP2L), Nuclear Factor 45 (NF45), NF90, RuvB-Like 1 (RUVBL1)) (127). A quantitative phosphoproteomic study showed that loss of *O*-GlcNAcylation in OGT null cells affects phosphorylation of cell cycle and DDR proteins including ATM and Checkpoint Kinase 1 (CHK1). Notably, hypo-*O*-GlcNAcylation increases activating phosphorylation events on ATM at Ser^1981^ and on its downstream substrates p53, H2AX, and CHK2 (128). In irradiated cells, OGT is essential for DNA damage repair, recovery from checkpoint arrest, blockage onset of cellular senescence, and cell survival (126, 129, 130). Quantitative proteomic profiling of irradiated MCF-7 breast cancer cells revealed a strong regulation of chromatin modification and organization and DDR-associated factors upon alteration of *O*-GlcNAcylation (126). The H3K27 methyltransferase (HMT) Enhancer of Zeste 2 (EZH2) of Polycomb Related Complex 2 (PRC2) is considered a potential link between *O*-GlcNAcylation levels and irradiation induced DDR. EZH2-mediated histone methylation is found to promote DSB repair (131) and its Ser^76^ and Ser^84^ *O*-GlcNAcylation increases its stability and its HMT activity (132, 133). Irradiated tumor xenografts treated with shOGT or alloxan OGT inhibitor displayed a decreased EZH2 level, persistent DNA damages, a reduced proliferation, and an increased senescence. PUGNAc-induced elevation of *O*-GlcNAcylation or feeding animals with GlcNAc protects tumors against irradiation (126). Moreover, upon irradiation, *O*-GlcNAcylated H2B at Ser^122^ interacts with Nijmegen Breakage Sydrome 1 protein a member of MRE11-RAD50-NBS1 (MRN) complex, and promotes its accumulation at DSB where it normally acts as a bridge spanning the broken ends (129).

Other study showed contradictory results and point out OGT as a negative regulator to limit the expansion of DDR signaling in response to irradiation. Notably, in irradiated HeLa cervical cancer cells, OGT *O*-GlcNAcylates H2AX at Ser^139^ and Mediator of DNA Damage Checkpoint 1 (MDC1), and restrains the expansion of the DSBs-induced phosphorylation from the DNA damage sites. Thus, depletion of OGT in these cells does not affect DSB repair but prolongs the G_2_/M checkpoint, delays cell cycle recovery, and reduces cell viability following DNA damages (37).

## Conclusion and perspectives: *O*-GlcNAcylation as a therapeutic target to overcome resistance to anti-cancer therapies

Since its discovery in the 1980s (134, 135), the community have extensively proven the implication of *O*-GlcNAcylation in the etiology of chronic human diseases such as metabolic, neurodegenerative, cardiovascular diseases and cancer (6, 136, 137) but the role of the sugar in the resistance to various anti-cancer therapies has arisen more recently. Although many molecular mechanisms remain to be elucidated, targeting *O*-GlcNAcylation emerges as a promising strategy to impede cancer resistance. The current development of metabolic inhibitors, inhibitors of HBP and *O*-GlcNAcylation enzymes, RNA aptamers and nanobodies appear as promising tools and must be investigated in the light of cancer therapies to prevent or obliviate chemoresistance. The reprogramming of glucose and amino acid metabolisms provides energy and metabolites to cancer cells, including substrates for *O*-GlcNAcylation, to support growth, progression, metastasis, and resistance to anti-cancer therapies. Thus, a first level of action is to use metabolic inhibitors to modulate enzymes and transporters involved in nutrient metabolisms. The 2-deoxy-D-glucose (2DG) and dapagliflozin to target glucose metabolism, and GRASPA and ERY001 to target amino acid metabolism are currently under investigation in clinical trials (138). Direct targeting of HBP enzymes could be an alternative strategy. Thus, some inhibitors more or less specific for the rate-limiting GFAT (*i.e.* DON, azaserine and RO0509347), PGM3 (*i.e.* FR054) or UDP-N-Acetylglucosamine Pyrophosphorylase 1 (UAP1) (*i.e.* UTP a,b-methylenebisphosphonate analogue (meUTP) and GAL-012) are currently used (5). Notably, FR054 PGM3 specific inhibitor appears to have an *in vivo* antitumor efficacy by inhibiting the HBP flux (139). A third considered strategy is to use OGT or OGA inhibitors to directly restore the homeostasis of *O*-GlcNAcylation (5). The small molecule OSMI-1 is a highly specific OGT inhibitor of the quinolinone-6-sulfonamide (Q6S) class. Despite being membrane permeable and relatively large, having low aqueous solubility and inducing toxicity limiting its activity *in cellulo* (140), OSMI-1 could still have therapeutic potential since it synergistically enhances some cancer therapies (*i.e.* TRAIL and DOX)-induced apoptosis *in vivo* in tumor xenograft models (73, 113). The aminothiazoline derivative Thiamet-G is the most widely used OGA inhibitor *in vitro* and *in vivo* due to its stability, selectivity, oral bioavailability, and ability to cross the blood-brain barrier (141). Chronic elevation of *O*-GlcNAcylation by prolonged treatment with Thiamet-G (more than 5 months) was shown nontoxic *in vivo* in mice (142). We recently shown the efficiency of Thiamet-G to improve global *O*-GlcNAcylation levels and 5-FU response in murine carcinogen-induced CRC tumors (34). In addition, other selective OGA inhibitors, MK-8719 (143) and ASN120290 (previously known as ASN-561) (144) have obtained the orphan drug designation by the FDA for the treatment of the progressive supranuclear palsy (PSP) in 2016 and 2018 respectively. However, modulating global cellular *O*-GlcNAcylation levels have limits in term of therapeutic strategy. More than 5000 proteins have been identified as *O-*GlcNAcylated to date. Analysis of this *O*-GlcNAcome revealed the diversity of cellular mechanisms in which it is involved (145). In this way, global regulation of *O*-GlcNAcylation levels could affect proteins essential to cell/tissue physiology and induces severe side effects or induces other pathologies (6). To overcome this limitation, tools to implement a fourth strategy are under development which consists on targeting the *O*-GlcNAcylation of specific protein(s) of interest. Several writing and erasing *O*-GlcNAcylation engineering have been developed and some can allow the glycosylation/deglycosylation of a protein without the need for prior identification of glyco-sites (146). Notably and very recently, a series of modulated RNA aptamers (147) and nanobody-OGT/OGA (148–150) able to direct these enzymes to a specific target protein of interest have been designed and delivered to living cells. These new approaches seem promising but require further studies to prove their efficiency and explore their possible *in vivo* applications.

## Author contributions

NV and IEB co-conceived, co-organized and co-wrote the manuscript. NV prepared the figures. All authors have read and approved the final manuscript.

## Funding

This work was supported by the “Ligue Contre le Cancer/Comité du Nord/Comité de la Somme”, the University of Lille and the “Center National de la Recherche Scientifique”.

## Acknowledgments

We thank Dr Stéphan Hardivillé and Dr Isabel Gonzalez Mariscal for proofreading the review.

## Conflict of interest

The authors declare that the research was conducted in the absence of any commercial or financial relationships that could be construed as a potential conflict of interest.

## Publisher’s note

All claims expressed in this article are solely those of the authors and do not necessarily represent those of their affiliated organizations, or those of the publisher, the editors and the reviewers. Any product that may be evaluated in this article, or claim that may be made by its manufacturer, is not guaranteed or endorsed by the publisher.

## Glossary

**Table:**

5-FU	5-fluorouracil
ATM	Ataxia Telangiectasia Mutated
Bid	BH3 interacting domain death agonist
BTZ	bortezomib
CHOP	C/EBP Homologous Protein
CRC	colorectal cancer
DDR	DNA damage response
DOX	doxorubicin
DSB	DNA double-strand break
ER	endoplasmic reticulum
ERRFI1	ERBB Receptor Feedback Inhibitor 1
ERa	Estrogen Receptor alpha
EZH2	Enhancer of Zeste 2
FDA	Food and Drug Administration
FOX	Forkhead box
GEM	gemcitabine
GFAT	Glutamine Fructose-6-phosphate Amidotransferase
GPX4	Glutathione Peroxidase 4
H2AX	H2A histone family member X
HBP	hexosamine biosynthetic pathway
HSP	Heat Shock Protein
IR	ionizing radiation
IRE1a	Inositol- Requiring Enzyme 1a
MCL	mantle cell lymphoma
NF-kB	Nuclear Factor-Kappa
NRF1	Nuclear Respiratory Factor 1
NRF2	Nuclear factor erythroid-2-Related Factor 2
OGA	O-GlcNAcase
O-GlcNAc	O-linked b-Nacetylglucosamine
OGT	O-GlcNAc Transferase
OS	osteosarcoma
PD-L1	Programmed Death-Ligand 1
PERK	Protein Kinase RNA-like kinase
PGM3	Phosphoacetylglucosamine Mutase 3
PKM2	Pyruvate Kinase M2
Pol	DNA Polymerase
PTM	post-translational modification
ROCK2	Rhoassociated Coiled-coil forming protein Kinase 2
ROS	reactive oxygen species
sCLU	secretory Clusterin
Ser	serine
Thr	threonine
TRAIL	TNF-elated Apoptosis Inducing Ligand
TRAIL-R	TRAIL Receptor
TS	Thymidylate Synthase
UDP-GlcNAc	uridine disphosphate Nacetylglucosamine
UPR	unfolded protein response
YAP	Yes-Associated Protein
